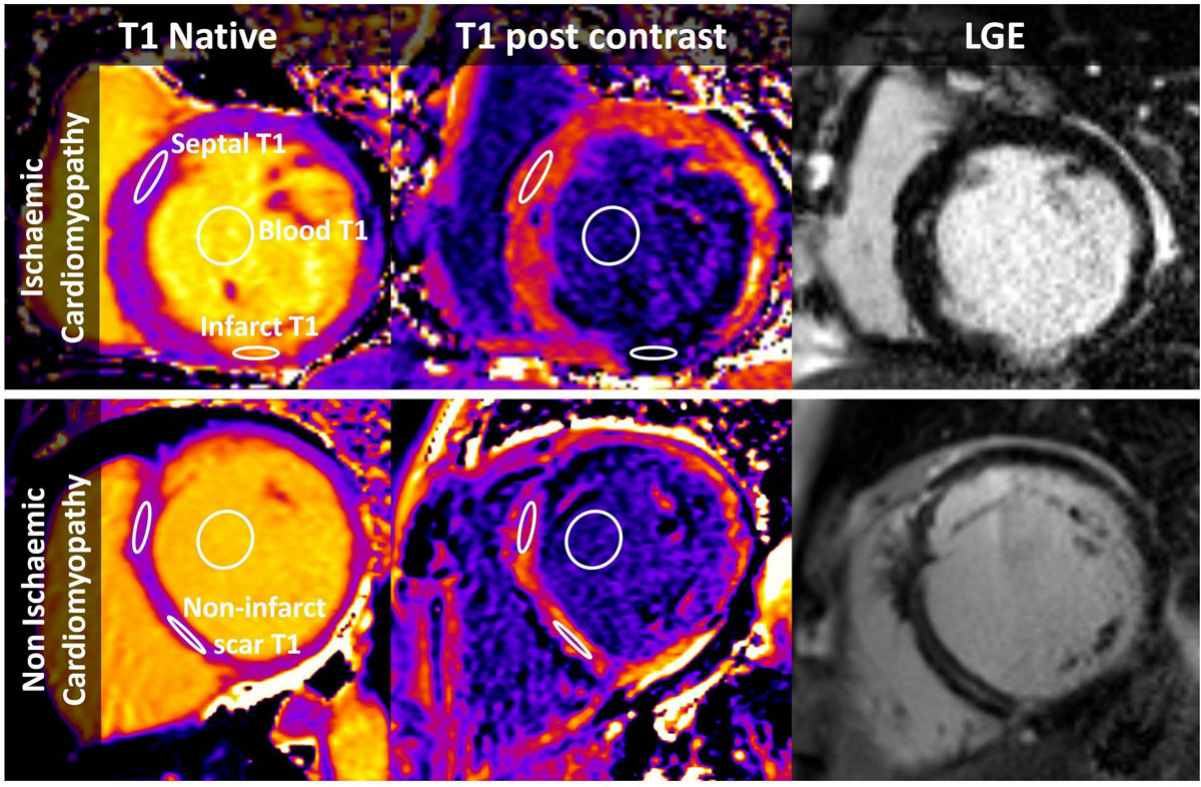# Insights from T1 mapping in heart failure

**DOI:** 10.1186/1532-429X-17-S1-P328

**Published:** 2015-02-03

**Authors:** James Waller, Ausami Abbas, Mohammed ElRefai, Jennifer Bryant, Peter J Cowburn, Peter J Weale, Stephen Harden, Charles Peebles, James Shambrook, Andrew S Flett

**Affiliations:** 1 Cardiology, University Hospital Southampton, Southampton, UK; 2 Siemens Healthcare, Camberley, UK

## Background

T1 mapping is a rapidly evolving field and is emerging as a novel quantitative tissue characterisation technique with potential applications in fibrosis, oedema, fat, iron and other patho-physiology. Its significance in heart failure is not yet fully explored but has the potential to add insights into disease processes and aid in refining diagnosis.

## Methods

Sixteen healthy subjects and 35 patients with heart failure awaiting guideline indicated CRT or primary prevention ICD (16 non ischemic cardiomyopathy (NICM), 19 ischemic cardiomyopathy (ICM)) were scanned on a 1.5T Magnetom Avanto (Siemens Healthcare, Erlangen) using a MOLLI (investigational WIP#448, 5:3:3 acquisition scheme, motion correction and automatically generated T1 map). In all cases on the basal short axis slice, a single, slender ROI was placed in the mid wall of the septum with meticulous attention to avoid partial volume of blood. In addition, the T1 was measured within scar when present on the T1 map (confirmed on the corresponding basal LGE image). When a contemporaneous haematocrit was available (n=24), the extracellular volume (ECV) was calculated using standard bolus acquisition methods.

## Results

Baseline characteristics are presented in table 1 with associated T1 and ECV values. Mean healthy myocardial T1 (1006±36ms) was shorter than ICM T1 (1058±35ms) and NICM T1 (1065±48) p=0.0002 and p=0.0007 respectively. There was no difference between NICM and ICM T1 nor ECV (0.29±0.04 and 0.29±0.05 respectively). The T1 in NICM scar tissue was significantly shorter and less variable (1144±67ms) than in ICM scar (1250±143ms), p=0.028. NICM scar ECV is less than ICM scar ECV (0.4±0.13 vs 0.63±0.20), p=0.024. Mass and indexed left atrial area (LAAi) correlated with NICM septal T1 ((r2 = 0.4, p=0.010 and r2=0.311, p=0.02) but not with ICM septal T1. Mass negatively correlated with ICM scar ECV (r2=0.36, p=0.035) but not with NICM scar ECV.

**Table 1:** baseline, T1 and ECV characteristics

	NICM	ICM	p
n (male)	16(11)	19(17)	0.13

Age	67±9	62±8	0.12

BMI	30±6	29±5	0.45

LAAi	15±8	15±4	1.00

EF%	26±8	29±7	0.18

QRS(ms)	153±27	132±25	0.02*

Mass	183±50	177±42	0.65

Septal T1	1065±48	1058±35	0.60

Scar T1	1144±67	1250±143	0.03*

Septal ECV	0.29±0.04	0.30±0.05	0.85

Scar ECV	0.40±0.13	0.63±0.20	0.02*

## Conclusions

We have demonstrated that in both ischaemic and non-ischaemic cardiomyopathy that T1 in the septum is elevated, even when it would be categorised as "normal" by LGE. We also show that the different pathogenesis of these cardiomyopathies result in "scar" in the non-ischaemic case exhibiting a shorter T1 and smaller ECV than ischaemic scar. In ischaemic scar there is a negative correlation with mass. There is a correlation of both mass and atrial size to septal T1 in the non-ischaemic group which suggests that T1 may be a surrogate for disease progression in this diffuse disease process.

## Funding

N/A.

**Figure 1:**